# Alkali metal cations enhance CO_2_ reduction by a Co molecular complex in a bipolar membrane electrolyzer

**DOI:** 10.1098/rsta.2023.0268

**Published:** 2024-09-23

**Authors:** Bhavin Siritanaratkul, Mohammad Danish Khan, Eileen H. Yu, Alexander J. Cowan

**Affiliations:** ^1^ Department of Chemistry, Stephenson Institute for Renewable Energy, University of Liverpool, Liverpool L69 7ZF, UK; ^2^ Department of Chemical Engineering, Loughborough University, Loughborough LE11 3TU, UK

**Keywords:** carbon dioxide reduction, bipolar membrane, zero-gap electrolyzer, molecular catalyst, cation

## Abstract

The electrochemical reduction of CO_2_ is a promising pathway for converting CO_2_ into valuable fuels and chemicals. The local environment at the cathode of CO_2_ electrolyzers plays a key role in determining activity and selectivity, but currently some mechanisms are still under debate. In particular, alkali metal cations have been shown to enhance the selectivity of metal catalysts, but their role remains less explored for molecular catalysts especially in high-current electrolyzers. Here, we investigated the enhancement effects of cations (Na^+^, K^+^, Cs^+^) on Co phthalocyanine (CoPc) in a state-of-the-art reverse-biased bipolar membrane electrolyzer. When added to the anolyte, these cations increased the Faradaic efficiency for CO, except in the case of Na^+^ in which the effect was transient, but the effects are convoluted with the transport process through the membrane. Alternatively, these cations can also be added directly to the cathode as chloride salts, allowing the use of a pure H_2_O anolyte feed, leading to sustained improved CO selectivity (61% at 100 mA cm^−2^ after 24 h). Our results show that cation addition is a simple yet effective strategy for improving the product selectivity of molecular electrocatalysts, opening up new avenues for tuning their local environment for CO_2_ reduction.

This article is part of the discussion meeting issue ‘Green carbon for the chemical industry of the future’.

## 1. Introduction

The electrochemical reduction of CO_2_ is intensively studied as a pathway for converting waste CO_2_ into valuable fuels and chemicals [1–3]. Using gas diffusion electrodes to increase the transport of CO_2_ to the catalyst, high performances (current densities and selectivities) have been achieved. Typically, reports use devices that generate a local alkaline condition at the cathode due to the presence of an anion exchange membrane and/or an alkaline catholyte. This suppresses the competing H_2_ evolution reaction, giving rise to high CO_2_ reduction Faradaic efficiencies [4,5]. However, the alkaline condition presents an obstacle, in that the CO_2_ feed directly reacts with hydroxide, converting to (bi)carbonate, which leads to low CO_2_ utilization levels and thus incurring an energy cost to regenerate the (bi)carbonates into usable gaseous CO_2_ [6].

Therefore, a recent focus of the field has been on electrolyzers with acidic environments at the cathode, with an aim towards increasing the CO_2_ utilization efficiency [7]. Bipolar membrane (BPM) electrolyzers in the reverse-bias configuration, i.e. with the cation exchange layer towards the cathode, are promising devices due to high CO_2_ utilization. Within the BPM, at the interface between the cation and anion exchange layers, H_2_O dissociation occurs, and H^+^ is transported to the cathode while OH^−^ is transported to the anode. This prevents carbonate formation at the cathode and subsequent crossover to the anode. Bipolar membranes also have additional benefits of allowing the cathodes and anodes to operate at different pHs at steady state, and the decreased product crossover. The challenge with reverse-biased BPM electrolyzers and more generally acidic environments at the cathode is the identification of suitable catalysts and structures that can achieve high enough Faradaic efficiencies for CO_2_ reduction. Approaches to alleviating this problem include inserting a thin electrolyte layer, which can be buffering or non-buffering, between the cathode and the BPM to increase the local pH under operating conditions [8,9], and decreasing the acidity of the cation exchange layers [10]. Alternatively, we demonstrated that molecular catalysts such as [Ni(cyclam)]^2+^ (cyclam = 1,4,8,11-tetraazacyclotetradecane) and [Mn(bpy)(CO)_3_Br] (bpy = 2,2′-bipyridine), which are inherently active in acidic conditions, can perform CO_2_ reduction selectively in zero-gap BPM electrolyzers [11,12].

Recently, we also reported CoPc to be selective for CO_2_ reduction to CO in a zero-gap BPM electrolyzer with an acidic cathode environment, and presented preliminary results showing that the selectivity was improved in the presence of K^+^ at the cathode [13]. The presence of cations, whether alkali metal cations (Na^+^, K^+^, Cs^+^) or alkylammonium cations (tetraethylammonium, poly(dimethyl diallylammonium)), has been shown to be crucial for enabling CO_2_ reduction on metal catalysts (Ag, Au, Cu), particularly in low bulk pH conditions [14–19]. Various mechanisms have been proposed for these activation effects, mainly: (i) hydrolysis of hydrated cations leading to lower local pHs and a higher local CO_2_ concentration, (ii) the generation of a greater electric field, which stabilizes the adsorbed intermediate CO_2_* and hinders migration of hydronium towards the cathode, due to the presence of a compact cation layer, and (iii) that the alkali cations can directly coordinate with and stabilize the adsorbed CO_2_*.

At molecular catalysts, there are also reports of cation enhancement effects, but they are mostly studied in homogeneous, low-current configurations, and it is not clear whether the mechanisms of selectivity enhancement are the same as for metal catalysts [20]. In an early study on Co and Ni tetraazamacrocycle complexes, cations changed the binding constant for CO_2_, with smaller cations leading to stronger binding [21]. Divalent cations (e.g. Mg^2+^, Ca^2+^) were reported by Bhugun *et al*. to increase the selectivity of homogeneous Fe porphyrin catalysts in DMF [22]. Wang *et al*. reported that Mg^2+^ enhances the performance of Co phthalocyanine (CoPc), possibly due to stabilizing adsorbed CO_2_* by generating a CoPc-CO_2_-Mg^2+^ complex [23]. Direct coordination of Mg^2+^ to a CO_2_* intermediate on a Mn bipyridine complex was also reported by Sampson *et al*. [24]. Very recently a similar mechanism has also been proposed by Sato *et al*. for K^+^ coordinating to CO_2_* at another Co molecular catalyst (Co tetrapyridino-porphyrazine) [25].

**Figure 1.**
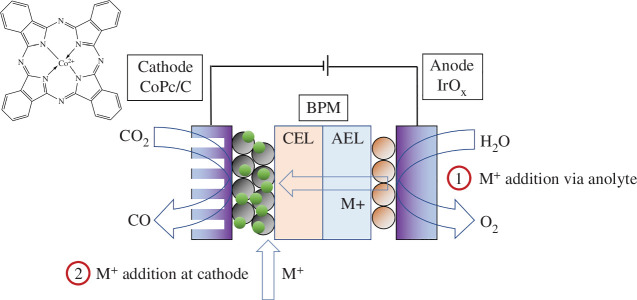
Diagram of the reverse-bias bipolar membrane (BPM) electrolyzer, with the cation exchange layer (CEL) directly in contact with the cathode (Co phthalocyanine supported on carbon (CoPc/C) on a gas diffusion layer), and the anion exchange layer (AEL) in contact with the anode (IrO_*x*_). M^+^ is Na^+^, K^+^ or Cs^+^, added either as (1) MOH in the anolyte, or (2) as MCl dropped onto the cathode before assembly.

Here, we investigate the effects of alkali metal cations on the performance of a Co molecular catalyst (CoPc) in a zero-gap bipolar membrane electrolyzer. A schematic of the cell is shown in figure 1. The cations can be added in two ways: (i) into the anolyte, capitalizing on the unintended cation crossover to the cathode, or (ii) directly deposited onto the cathode. Through this study, we identify that the direct addition of metal chloride salts to the cathode, while using a pure water anolyte, is an effective route to achieve increased selectivities for CO production with significantly improved device stabilities.

## 2. Experimental methods

### (a) Materials

The following chemicals were used as received: Co(II) phthalocyanine (Sigma Aldrich, 97%), Ensaco 350G carbon powder (Imerys), NaOH (Fisher, 98.7%), KOH (Sigma Aldrich, ACS reagent >85%), CsOH monohydrate (Sigma Aldrich, 99.95%), NaCl (Fisher, 99.5%), KCl (Fisher, 99%), CsCl (Sigma Aldrich, 99.5%), isopropanol (Sigma Aldrich, 99.5%), carbon paper (Sigracet 39BB), Ir(Iv) oxide (Alfa Aesar, 99.99%), Fumasep FBM bipolar membrane, Nafion solution (Sigma Aldrich, 5% in a mixture of a lower aliphatic alcohols and water) and CO_2_ (BOC, CP grade). The BPM was delivered and stored in 1.0 M NaCl, and rinsed and kept in pure H_2_O for 1 h before starting an experiment.

### (b) Electrode fabrication

The CoPc/C cathode was prepared by spraying a suspension of dispersed CoPc onto carbon paper (Sigracet 39BB). First, the carbon support (Ensaco, 1 mg cm^–2^) was dispersed in isopropanol (1 mL cm^–2^) for 1 h. Then CoPc (0.2 mg cm^–2^) was added to the suspension, then sonicated for a further 1 h. The suspension was stirred overnight at room temperature, then Nafion solution (1.2 μL cm^–2^) was added. The suspension was sprayed onto carbon paper held at 40°C on a hot plate using an airbrush with N_2_, then left to dry under ambient conditions. The IrO_*x*_ anode was prepared using a sono-sprayer (SonoTek), with IrO_*x*_ loading of 1 mg cm^–2^ in isopropanol.

### (c) Electrochemistry

Electrochemical measurements were made in an electrolyzer (Dioxide Materials, cathode plate (stainless steel) 5 cm^2^, anode plate (titanium) 9 cm^2^, serpentine flow channels), using an Ivium Vertex potentiostat. The inlet CO_2_ stream was controlled by a mass-flow controller (Buerkert) at 80 sccm, and passed through a water bubbler at room temperature. The anolyte was circulated by a peristaltic pump (15 mL min^–1^). Experiments were conducted at ambient pressure and temperature (20–22°C).

### (d) Product detection

Products were detected by connecting the cathode outlet of the electrolyzer to a gas chromatograph (Varian CP-4900), with a thermal conductivity detector, a Molsieve column and Ar as the carrier gas.

CO Faradaic efficiency (FE) calculation


$$CO\;\;FE\;\left(\%\right)\;=\;\frac{Current\;towards\;CO\;production}{Total\;current}\;=\frac{xv_{out}\;P/RT}{JA/2F}\;\times \;100,$$

where *F* is Faraday’s constant, *J* is current density, *A* is electrode area, *ν*_out_ is the total volumetric outlet flow rate, *x* is the outlet molar fraction of CO, *P* is the pressure, *R* is the gas constant and *T* is the temperature.

### (e) Characterization

Scanning electron microscopy (SEM) and energy dispersive X-ray (EDX) observations were made using a Hitachi SEM S4800 at 20 kV. Inductively coupled plasma (ICP) spectrometry was conducted using a Perkin Elmer nexION 2000 ICP-MS, after digesting the samples in HNO_3_(conc). X-ray photoelectron spectroscopy (XPS) was performed using a Thermo Scientific K-Alpha instrument with Al Kα Xray (1486.6 eV) source on 400 × 400 μm^2^ spot size. The energy calibration was performed by using C 1s peak at 284.8 eV.

## 3. Results

In these studies, we use a CoPc catalyst on the cathode, supported on carbon powder and sprayed onto carbon paper, the membrane is a commercial Fumasep BPM, in reverse-bias configuration (with the cation exchange layer directly contacting the cathode in a zero-gap configuration) and the anode is IrO_*x*_/carbon paper, a standard water oxidation catalyst.

First, we demonstrate the effects of Na^+^, K^+^ or Cs^+^ on CoPc, when the cations are introduced into the anolyte. In addition to H^+^ and OH^–^ transport (through the CEL and AEL, respectively, following water dissociation at the interface of the two layers), it is important to also consider the potential for transport of alkali cations through the membrane through co-ion crossover [26,27].

**Figure 2.**
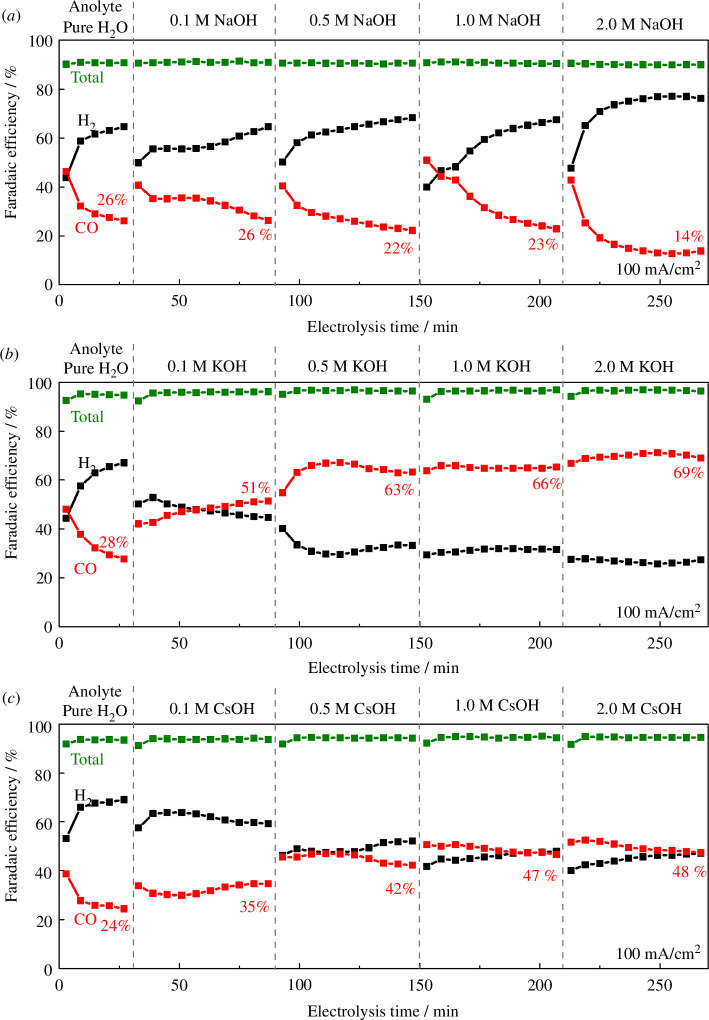
Time courses of Faradaic efficiencies to H_2_ (black) and CO (red) at 100 mA cm^–2^, with a CoPc/C catalyst in a reverse-bias zero-gap BPM electrolyzer, with additions of (*a*) NaOH, (*b*) KOH and (*c*) CsOH into the anolyte. The anode was IrO_*x*_, and the anolyte was initially pure H_2_O. The cathode feed was CO_2_ saturated with H_2_O vapour, at 80 sccm. Other conditions: Fumasep FBM bipolar membrane, room temperature.

Here, to benchmark activity in the absence of deliberately added alkali metal cations, we conducted two-electrode potentiometry at 100 mA cm^–2^, initially with pure H_2_O (Milli-Q, Type 1, Resistivity > 18.2 MΩ cm^–1^) as the anolyte and a humidified CO_2_ gas supply (80 sccm) to the cathode. After 30 min, we switched the anolyte to 0.1 M MOH (where M is Na, K or Cs), then resumed measurement for 1 h. This was further repeated with 0.5, 1.0 and finally 2.0 M MOH, for 1 h at each concentration, and the time courses of the Faradaic efficiencies are shown in figure 2.

Figure 2 shows that regardless of the nature of the anolyte the only product of the electrolysis is CO and H_2_ with the sum of the Faradaic efficiencies of these accounting for all charge passed. With a pure water anolyte, the CO Faradaic efficiency drops from approximately 50% for CO to <30% within 30 min as has previously been observed [13], and we previously attributed the change in selectivity to an increase in hydration and flooding of the electrode partially hindering CO_2_ transport.

When K^+^ or Cs^+^ is added to the anolyte, the CO selectivity was increased and the device stability was improved. Initially upon switching to the 0.1 M MOH salt (M = Cs, K), we observed a slow increase in CO yield, which is in line with the expectation of transport across the anolyte leading to cations at the cathode controlling activity. As the MOH salt concentration in the anolyte is initially increased we see increases in the CO Faradaic efficiency, but this was almost saturated at 0.5 M concentration, and >0.5 M MOH anolyte did not appreciably increase the CO Faradaic efficiency further. Using MOH salts in the anolytes we reach a plateau of 63–69% and 42–48% for K^+^ and Cs^+^, respectively, for the Faradaic efficiency for CO production.

By contrast, although the use of an NaOH anolyte resulted in an initial increase in CO Faradaic efficiency upon restarting the experiment, the effect was found to be transient and selectivity subsequently decreased through each 1 h segment (figure 2). The changes in selectivity are unlikely to be due to the stoppage/restart of the electrolyzer, since we have shown previously that the selectivity of this catalyst and cell configuration was essentially unchanged after a pause [13]. The cell voltages are shown in the electronic supplementary material, figure S1. Upon addition of hydroxides into the anolyte, the cell voltage decreased by approximately 0.3–0.4 V, which is anticipated due to improved water oxidation kinetics at the anode at high pH and lower solution resistance in the anolyte.

We next verified and quantified the amount of cation crossover after anolyte addition experiments. Using a fresh CoPc cathode for each experiment, we ran the electrolyzer with four different anolytes (pure H_2_O, 1 M NaOH, 1 M KOH or 1 M CsOH) at 100 mA cm^–2^ for 1 h, then collected the used cathodes for analysis. After these experiments, there were no visible salt deposits at the cathode plate of the electrolyzer, indicating that although the cations did cross over, the amount did not cause salt precipitation and blockage of the gas channels on this timescale.

**Figure 3.**
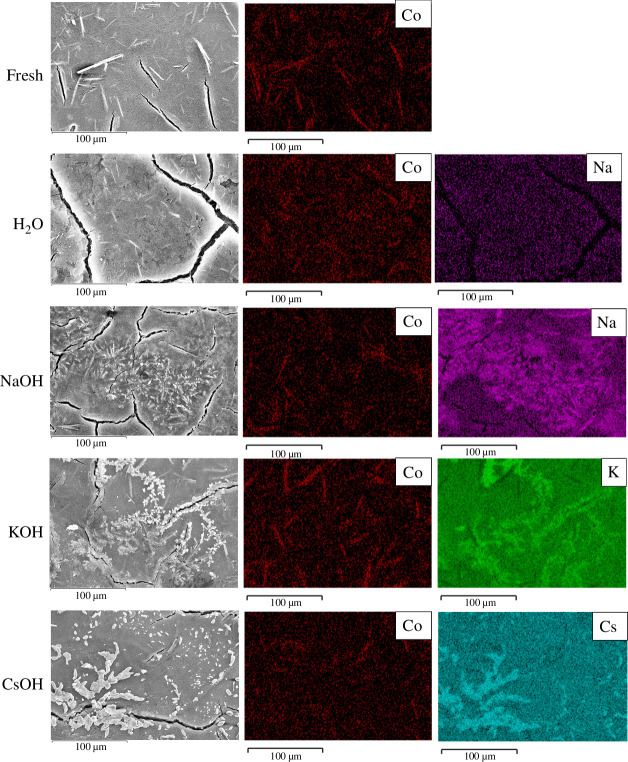
Scanning electron microscopy (SEM) images and the corresponding energy dispersive X-ray (EDX) mapping of fresh and used CoPc/C cathodes, with the anolyte as pure H_2_O, 1 M NaOH, 1 M KOH or 1 M CsOH, operated at 100 mA cm^−2^ for 1 h. The anode was IrO_*x*_. The cathode feed was CO_2_ saturated with H_2_O vapour, at 80 sccm. Other conditions: Fumasep FBM bipolar membrane, room temperature.

The SEM images and the corresponding EDX mapping of the fresh and used cathodes are shown in figure 3. As a semi-quantitative measure, the atomic ratios of the cation and Co are given in table 1. In runs using pure H_2_O as anolyte, small amounts of Na were observed, which was much lower than when using 1 M NaOH anolyte. These small Na contaminations are due to residual Na from the storage solution of the BPM. Although care was taken to exchange the Na^+^ out of the BPM by equilibrating in pure H_2_O before use, there were still tightly bound Na^+^ from the NaCl storage solution that presumably would only be removed under reverse-bias operation. In the cation-containing anolyte runs, the corresponding cations were clearly deposited onto the cathode. EDX mapping cannot identify the nature of the anion but it is anticipated that bicarbonate salts will have formed on the cathode surface. Ir^4+^ from IrO_*x*_ dissolution could also potentially be a charge carrier and influence the selectivity [28], but we did not observe any Ir signals from EDX measurements regardless of the anolyte.

**Table 1.** Amount of deposited cations, quantified by EDX mapping and ICP spectrometry, for different anolytes after 100 mA cm^−2^ for 1 h.

anolyte	EDX M/Co atomic ratio	ICP deposited M amount / μmol cm^−2^
pure H_2_O	Na 2.6	Na 7.0
1 M NaOH	Na 22.7	Na 48.8
1 M KOH	K 24.7	K 21.9
1 M CsOH	Cs 27.7	Cs 16.4

ICP spectrometry was also used to quantify the deposited amount of cations, and the results are shown in table 1. The discrepancy in the trends of EDX and ICP results is attributed to the EDX signals arising from near the surface (approx. μm scale), whereas for the ICP measurement the whole electrode was submerged in a solution to leach out all of the deposited salt. The deposited salt amounts, which are a measure of the cation transport across the membrane, accounted for 0.2–1.3% of the total ionic charge passed during the chronopotentiometry measurement, and the balance will be via the transport of H^+^/OH^−^ generated by water dissociation in the reverse-biased BPM.

The results in figure 2 demonstrate that the CoPc cathode in the reverse-biased BPM is sensitive to the nature of the MOH anolytes that are used, but the mechanism of differing behaviour, with K^+^ and Cs^+^ cations improving activity while Na^+^ only provides minimal long-term effect, is not clear. The crossover of K^+^ through a BPM has been previously reported with an increase in CO selectivity at an Ag cathode occurring, but the mechanism of enhancement was not examined [29].

One possible reason for selectivity enhancement in the presence of cations is an increased local pH at the cathode. When the cathode is held at a highly negative potential, alkali cations can accumulate within the double layer due to migration, and this lowers the local [H^+^], thus leading to lower hydrogen evolution reaction (HER) rates and higher CO selectivity. Recently, Sauve *et al*. quantified the extent of local pH changes using open circuit potential decay curves with Pt GDEs [30]. This method relies on Pt being a reversible catalyst for H^+^/H_2_ interconversion, therefore the open circuit potential immediately after stopping the current should report on the local pH generated at that current. The local pH could be higher than the bulk pH by up to 13 units, depending on the current density and concentration of the electrolyte. However, importantly, the alkali cation identity (Li^+^, Na^+^ or K^+^) did not lead to significant differences in local pH at the same concentration. From our results, substantial deposition of each cation was observed, yet the selectivity trend was markedly different for Na^+^ (compared with K^+^ or Cs^+^). Similarly, previous studies have conducted simulations of the local pH at the catalyst/electrolyte interface [17], and although modelling indicated that accumulation of Na^+^ at the outer Helmholtz plane (OHP) is less than that of K^+^ or Cs^+^, due to its larger hydrated radius, the cation identity did not have a significant effect on H^+^ migration (as measured from the mass transport-limited current in acid) [31]. We expect to investigate more detailed simulations in future work, taking into account the ion size via a Generalized Modified Poisson–Nernst–Planck formulation [32], and two-dimensional models that encompass the effects of pore sizes [33]. However, from these initial results we conclude that although the presence of alkali cations will have increased the local pH (as demonstrated below), this seems unlikely to be the only cause of selectivity enhancement. Of significance is a recent study using Co molecular catalysts (albeit in an anion-exchange membrane configuration) that also showed improvements in both selectivity and stability when KOTf was added directly to the cathode [25]. Here it is notable that although Na^+^ did also improve the selectivity of the catalyst, there was a smaller enhancement effect than observed with K^+^. Here it was proposed that the main mechanism of enhancement was from direct coordination of K^+^ with a CO_2_* intermediate and we propose a similar stabilization is possibly contributing to the activity here.

The preceding results with cation-supplemented anolyte clearly demonstrated the selectivity enhancement effect, but there were convolutions with the rate of cation transport through the membrane. In addition, a continuous crossover flux might not be sustained for steady-state operation, possibly requiring periodic water injections to remove salt build-up [34]. Therefore, we investigated an alternative addition method, by adding alkali metal cations directly to the cathode, and retaining pure H_2_O as the anolyte. In this configuration, although there is a concentration gradient of the cation between the cathode and the anode, cation crossover towards the anode should be minimal since the potential gradient should lead to retaining cations at the cathode.

**Figure 4.**
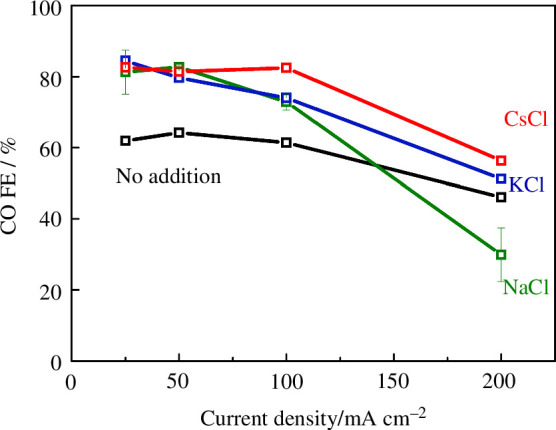
Faradaic efficiency for CO at various total current densities, using a CoPc/C cathode with no additional salts (black), NaCl (green), KCl (blue) or CsCl (red) added (100 μmol cm^−2^). The anode was IrO_*x*_, and the anolyte was pure H_2_O. The cathode feed was CO_2_ saturated with H_2_O vapour, at 80 sccm. Other conditions: Fumasep FBM bipolar membrane, room temperature. The results for no addition are from our previous report [13].

We chose chloride salts (NaCl, KCl or CsCl), since these salts were shown to be effective in improving the selectivity of metals such as Cu, and chloride ions do not lead to side reactions at the cathode. An aliquot of 1 M NaCl, KCl or CsCl solution was dropped onto a fresh CoPc/C electrode to give 100 μmol cm^−2^ loading, and left to dry before assembly into the electrolyzer. Figure 4 shows the CO Faradaic efficiencies at 25–200 mA cm^−2^, after 30 min at each current density. The cell voltages are shown in the electronic supplementary material, figure S2. After the measurements, there were no visible deposits on the cathode, indicating that the loaded salts were dissolved in the water (either generated from the CO_2_ reduction reaction or crossed over from the anolyte) and dispersed into the catalyst layer. All the cation additions increased the CO selectivity by up to 20 percentage points, except for Na at the highest current (200 mA cm^−2^), but we note that the selectivity at this current density was less stable than lower currents due to more rapid cathode flooding, which prevents access of CO_2_ to the catalyst (electronic supplementary material, figure S3). In principle, chloride anions could also have an effect on the performance, as was observed in some previous studies [35,36]. To deconvolute the effects of the anion, we also conducted a similar measurement with KHCO_3_ added, at the same molar loading (electronic supplementary material, figure S4). The product selectivities were very similar to the KCl addition run. While we cannot completely rule out the effects of the chloride anion, our results are consistent with the proposal that the cation addition is the main cause of activity enhancement.

**Figure 5.**
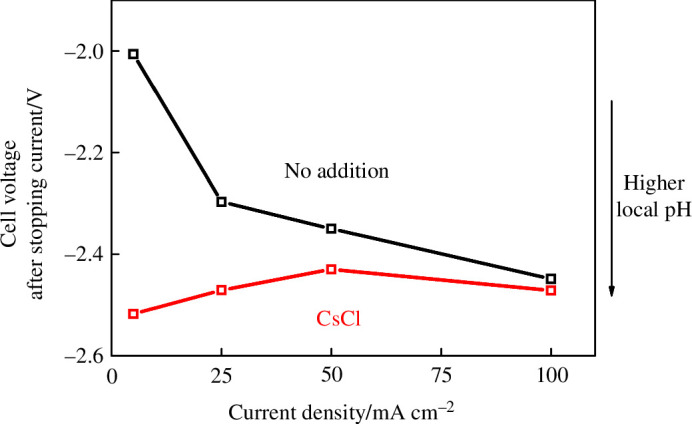
The OCP immediately after stopping the current, at a Pt/C (1.2 mg cm^−2^) electrode with no addition (black) and CsCl added (100 μmol cm^−2^, red). The anode was IrO_*x*_, and the anolyte was pure H_2_O. The cathode feed was Ar saturated with H_2_O vapour, at 20 sccm. Other conditions: Fumasep FBM bipolar membrane, room temperature.

To further elucidate the mechanism of selectivity enhancement, we probed the changes in local pH upon cation addition. Following the work of Sauve *et al*. [30] we substituted a Pt/C cathode into the same cell set-up and measured the open circuit potential (OCP), directly after applying a certain current density. The OCP at Pt should reflect the local pH reached at that current density (i.e. more negative = higher pH). Although the electrode structure differs slightly between Pt/C and CoPc/C, we used the same carbon paper support with similar loading, and qualitative trends should be applicable to the CoPc/C electrode. In a two-electrode measurement, the OCP reflects contributions from both the cathode and the anode, but since the anode is the same in both cases (IrO_*x*_ in pure H_2_O), any differences in the OCP can be attributed to the cathode environment. A current density was applied for 5 min, then the current was stopped and the OCP was monitored (electronic supplementary material, figure S5). The potential directly after stopping the current is shown in figure 5, for the bare Pt/C electrode and the one with CsCl loaded (100 μmol cm^−2^). In the no addition case, the OCP was more negative after a higher applied current, which is consistent with the more alkaline local pH when protons are consumed at a higher rate in a reduction reaction (both hydrogen evolution and CO_2_ reduction to CO are 2e^−^, 2H^+^ reductions). With CsCl added, the OCP was more negative compared with the no addition case after the same current, demonstrating that the additional alkali metal cation was effective in restricting H^+^ transport to the surface, leading to higher local pH and thus higher selectivity towards CO.

We next investigated the changes in CO_2_ binding upon cation addition. Yao *et al*. [37] demonstrated a method, using the relationship between the partial current to CO and the CO_2_ concentration. Following their method, we assume the following mechanism:


(2.1) $$\left[Co(II)Pc]\;+\;e^{-}\to [Co(I)Pc\right]$$

(2.2) $$\left[Co(I)Pc]\;+\;CO_{2}\overset{K_{co_{2}}}{⟷}[CO_{2}-Co(I)Pc\right]$$

(2.3) $$[CO_{2}-Co(I)Pc]\;+\;H^{+}\overset{RDS}{⟶}[COOH-Co(II)Pc].$$

**Figure 6.**
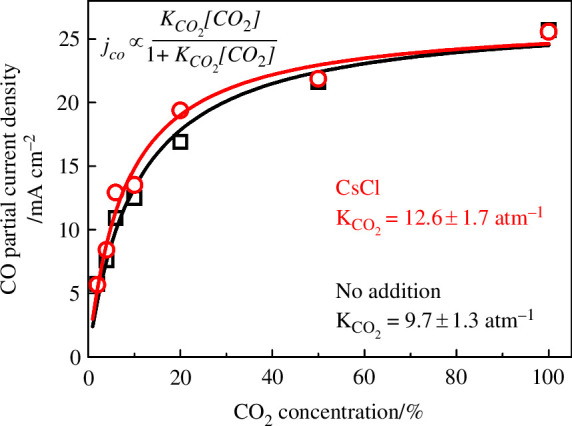
The CO partial current densities as a function of CO_2_ concentration, with no addition (black) or CsCl 100 μmol cm^–2^ added (red). The points are the measured results, and the lines are from fitting the partial currents to the shown relationship using ordinary least squares. The errors in the KCO_2_ fitting are estimated from the covariance calculated by the scipy package. The applied cell potential was −4.5 V for 10 min at each segment, and the order of experiments was: 6, 20, 100, 50, 10, 4, 2% for both experiments. The total flow was 100 sccm, with Ar balance.

The binding of CO_2_ to the one-electron reduced species Co(I)Pc is established as a pre-equilibrium prior to the rate-determining step (RDS), which is the subsequent reduction of CO_2_ at the CO_2_-bound Co(I)Pc species. Therefore, the partial current towards CO is proportional to the coverage of CO_2_-bound Co(I)Pc, which can be rewritten as:


$$j_{CO}\propto \frac{K_{CO_{2}}\;[{CO}_{2}]}{1+K_{CO_{2}}\;[{CO}_{2}]},$$

where *j*_CO_ is the partial current density towards CO, K_CO2_ is the binding constant for CO_2_ and [CO_2_] is the CO_2_ concentration. Figure 6 shows *j*_CO_ at a constant cell voltage of −4.5 V, under CO_2_ concentrations of 2–100% (atmospheric pressure, Ar balance). The measured *j*_CO_ could be fitted well with the above relationship, consistent with the proposed RDS. If the RDS were a different step, e.g. the CO_2_ binding step, this would have resulted instead in a linear relationship between *j*_CO_ and [CO_2_]. Upon CsCl addition (100 μmol cm^−2^), the *j*_CO_ in the low concentration range increased marginally, thus leading to only a small increase in the K_CO2_ (within the uncertainties of the fitting). Although we cannot completely rule out the effect of cations on the binding of intermediates, our results here suggest that the effect is small for CoPc and alkali metal ions. The degree of binding enhancement should be highly dependent on both the properties of the metal complex (e.g. ligand steric configuration) and the cation (e.g. compact alkali metal versus bulky quarternary ammonium), and future characterization complemented by computational studies is required to further investigate this mechanism.

**Figure 7.**
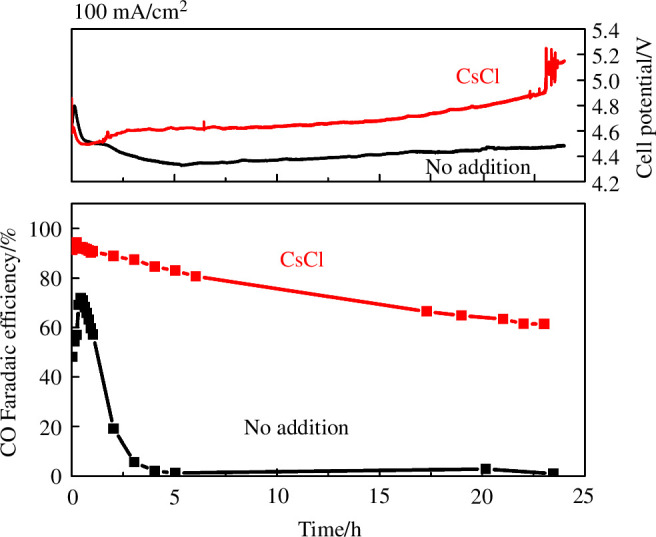
Stability of CoPc/C performance over 24 h, with no addition at the cathode (black) or CsCl 100 μmol cm^–2^ (red). The anode was IrO_*x*_, and the anolyte was pure H_2_O. The cathode feed was CO_2_ saturated with H_2_O vapour, at 80 sccm. Other conditions: Fumasep FBM bipolar membrane, room temperature.

Finally, we investigated the influence of cations on the stability of CO production by CoPc/C. We ran the electrolyzer at 100 mA cm^–2^, using pure H_2_O as anolyte, and compared the performance with and without CsCl addition for 24 h (figure 7). Without cation addition, CO selectivity rapidly declined from approximately 70% to <5% after 5 h, whereas with CsCl addition the initial CO selectivity was approximately 90%, and >60% was retained after 24 h. This is a marked improvement from our past studies using the same cathode and membrane, in which a 1 M KOH anolyte was used, there the CO selectivity dropped to approximately 30% after 24 h under the same conditions [13]. SEM/EDX of these 24 h used electrodes (electronic supplementary material, figure S6) show no significant aggregation of Co, and Cs was dispersed on the cathode in the case of CsCl addition. We also conducted XPS of the fresh and used cathodes (electronic supplementary material, figure S7). For all cases, the Co 2p spectra showed Co^2+^ assigned to CoPc (Co 2p_3/2_ at 780.9 eV and Co 2p_1/2_ at 796.4 eV), consistent with previous reports [13,38]. The C and N signals were also not significantly changed in the used electrodes. This demonstrated that after 24 h of reaction, intact CoPc species remained on the cathode. For the CsCl-added run, the used electrode still showed strong Cs signals, indicating that the added Cs^+^ was not significantly lost through the membrane even though the anolyte was pure H_2_O.

Our results show that the addition of high concentration of alkali metal cations, particularly K^+^ and Cs^+^, significantly enhanced the CO selectivity of CoPc. The enhancement is possibly due to both (i) direct coordination of the cation to a bound CO_2_* intermediate, lowering the reaction barrier, and (b) the electric field generated by these cations at the OHP hindering hydronium transport, thus lowering HER rates due to the higher local pH. Our mechanistic studies showed that with cation addition the extent of improvement of CO_2_ binding is small, but there was a significant increase in local pH, suggesting the latter mechanism is a significant factor. But why a selectivity difference between different alkali cations exists remains unknown. Based on our results, which show that pH is important, we also expect enhancement effects from cations that are not alkali metals, for example tetraalkyl ammonium (both in monomeric form in solution, and polymeric form as an overlayer on the catalyst) [15,16,39]. These bulky cations are expected to be weaker than alkali metal cations at coordinating to adsorbed CO_2_*. Therefore, if they were to show similar enhancement effects, this would indicate that the electrostatic hindrance of hydronium transport (thus HER suppression) is the main mechanism for CO selectivity enhancement on this molecular catalyst.

## 4. Conclusion

We investigated the effects of alkali metal cations on a molecular CO_2_ reduction catalyst, CoPc, at industrially relevant current densities (up to 200 mA cm^–2^), using a reverse-bias BPM electrolyzer suitable for scaling up due to high CO_2_ utilization. When using NaOH, KOH or CsOH solutions as the anolyte, the cations were able to cross over the BPM to the cathode. The presence of these cations at the cathode enhanced the selectivity towards CO when K^+^ or Cs^+^ was used, proposed to be due to both the stabilization of CO_2_ reduction intermediates [25] and the generation of a higher local pH at the cathode [30]. Mechanistic studies showed a significant pH increase upon Cs^+^ addition, but the increase in CO_2_ binding was marginal. Intriguingly, Na^+^ had only a transient positive effect on CO selectivity. The selectivity enhancement can also be achieved by simply directly adding chloride salts to the cathode, with the degree of enhancement in the order: Cs^+ ^> K^+ ^> Na^+^. This approach has the potential to enable prolonged operation of the device as the cations are likely to be retained at the cathode during operation. In an extended experiment (100 mA cm^–2^ for 24 h), CsCl addition greatly improved the stability, retaining >60% CO FE at 24 h, whereas without cation addition the selectivity rapidly declined after 5 h. Our results demonstrate that in addition to the known effects on metal catalysts, cations can also effectively enhance the performance of molecular catalysts for CO_2_ reduction at high current densities, expanding the scope for tuning their performance via tuning the local environment.

## Data Availability

Data is available from the depository [40]. Supplementary material is available online [41].
